# Node Identification Using Inter-Regional Correlation Analysis for Mapping Detailed Connections in Resting State Networks

**DOI:** 10.3389/fnins.2017.00238

**Published:** 2017-05-01

**Authors:** William S. Sohn, Tae Young Lee, Kwangsun Yoo, Minah Kim, Je-Yeon Yun, Ji-Won Hur, Youngwoo Bryan Yoon, Sang Won Seo, Duk L. Na, Yong Jeong, Jun Soo Kwon

**Affiliations:** ^1^Institute of Human Behavioral Medicine, Medical Research Center, Seoul National UniversitySeoul, South Korea; ^2^Department of Psychiatry, Seoul National University College of MedicineSeoul, South Korea; ^3^Department of Bio and Brain Engineering, KAISTDaejeon, South Korea; ^4^Department of Psychology, Chung-Ang UniversitySeoul, South Korea; ^5^Department of Brain and Cognitive Sciences, Seoul National UniversitySeoul, South Korea; ^6^Department of Neurology, Samsung Medical Center, Sunkyunkwan UniversitySeoul, South Korea; ^7^Neuroscience Center, Samsung Medical CenterSeoul, South Korea

**Keywords:** resting fMRI, node identification, subject-specific ROIs, Alzheimer's disease, connectomics

## Abstract

Brain function is often characterized by the connections and interactions between highly interconnected brain regions. Pathological disruptions in these networks often result in brain dysfunction, which manifests as brain disease. Typical analysis investigates disruptions in network connectivity based correlations between large brain regions. To obtain a more detailed description of disruptions in network connectivity, we propose a new method where functional nodes are identified in each region based on their maximum connectivity to another brain region in a given network. Since this method provides a unique approach to identifying functionally relevant nodes in a given network, we can provide a more detailed map of brain connectivity and determine new measures of network connectivity. We applied this method to resting state fMRI of Alzheimer's disease patients to validate our method and found decreased connectivity within the default mode network. In addition, new measure of network connectivity revealed a more detailed description of how the network connections deteriorate with disease progression. This suggests that analysis using key relative network hub regions based on regional correlation can be used to detect detailed changes in resting state network connectivity.

## Introduction

Modern neuroscience has shown that cognitive functions are composed of integrative processes coupled with dynamic interactions that can be distributed across a specific set of various brain regions (Barrett and Satpute, 2013; Power and Petersen, 2013; van den Heuvel and Sporns, 2013; Ester et al., 2015). In other words, the structural and functional connections in the brain, or the “connectome,” are thought to play a major role in behavior and cognitive performance (Park and Friston, 2013; Smith et al., 2015). Connectomics are of particular interest in the study of brain diseases because deficits in cognitive functions are thought to be representative of disruptions in cortical connectivity (Stam, 2014; Fornito et al., 2015). Many studies have shown that these disruptions in connectivity target specific functional networks in different neurological diseases and psychiatric disorders (Seeley et al., 2009). Therefore, detailed brain connectomes may provide a means to understand how changes in functional connectivity in specific networks or regions of the brain can characterize the symptoms and deficits in cognitive and behavioral functions (Crossley et al., 2014; Stam, 2014). Additionally, these patterns of neurodegeneration progress along large-scale networks (Seeley et al., 2009), supporting a “network degeneration hypothesis” that suggests that pathological brain changes occur primarily in vulnerable hub regions in distinct major brain networks (Drzezga et al., 2011; Li et al., 2016). Recent studies have proposed various models or mechanisms behind network degeneration (Zhou et al., 2012; Brier et al., 2014; Fornito et al., 2015), however these models are still being developed, and further studies are required.

An important step in network analysis is node selection. In brain network representations, nodes represent neural populations or brain regions that have shared structural or functional relevance (van den Heuvel and Sporns, 2013). The most common methods for node identification rely on anatomical or functional brain parcellations (Craddock et al., 2013; Smith et al., 2013). However, current methods are problematic because there are many different strategies and the results can based on the method used (Zalesky et al., 2010; Craddock et al., 2013; de Reus and van den Heuvel, 2013; Thirion et al., 2014). Additionally, template and group parcellations fail to address the issue of individual variability. This problem is illustrated in many studies which revealed distinct individual differences in the functional organization of individuals (Mueller et al., 2013; Finn et al., 2015; Hahn et al., 2015; Dubois and Adolphs, 2016), suggesting that a subject-specific approach may offer better representations of network connectivity (Chamberland et al., 2015; Li et al., 2015; Sohn et al., 2015; Wang et al., 2015). Finally, interpretation of the results is often limited by the way that nodes are defined. Traditional methods result in direct correlation values and connectivity matrices. These results are often interpreted by direct node to node connectivity, or as a whole with patterns or descriptive measures such as graph theory properties. With the exception of intra/inter network connectivity, it is difficult to classify and characterize specific types of connectivity within a connectome due to the nature of how nodes were defined. While this suitable to for distinguishing simple patterns of functional connectivity, detailed connectomes require a more comprehensive method of node selection. This is essential for developing existing models of functional changes which occur with various neurological disease.

We propose a new method that utilizes maximum connectivity among regions in a network to define functionally relevant nodes for resting state analysis. The idea of using maximum connectivity at the voxel level has been implemented in various other methods (Cole et al., 2010; Golestani and Goodyear, 2011). However the unique way in which this is implemented in our method allows for new definitions and characterizations of nodes and connectivity that in turn results in novel measures for subsequent connectivity and network analysis. We propose that these new measures will allow for more detailed and individualized connectomes, leading to in-depth analysis of functional connectivity. To illustrate the advantages of this method, we analyzed resting state fMRI scans of patients in various stages of Alzheimer's disease (AD). Using this method, we were able to identify well-known changes in patterns of connectivity in AD and in addition, create a more detailed description of network deterioration. We propose that this method can move beyond simple properties of brain network organization and more comprehensively describe the underlying pathophysiological mechanisms behind specific brain diseases.

## Methods

### Subject demographics

The patient dataset used in this study included 22 healthy controls (HC) matched to 65 subjects with aMCI and 25 patients with AD (Table 1). Of these subjects, 2 healthy controls, 26 aMCI patients and 1 AD patient were excluded due to age or MRI quality. Subjects under the age of 65 were excluded. Subjects were excluded based on MRI quality based on individual network reconstruction and movement with an average frame wise displacement over 0.2. Each patient underwent a comprehensive neurological examination, laboratory investigations, neuropsychological testing, and brain MRI. The patients' caregivers were also interviewed. Amnestic MCI was diagnosed based on the Clinical Research for Clinical Dementia of South Korea (CREDOS) criteria (Park et al., 2011), which is a modified version of Peterson's criteria (Petersen et al., 1997). Patients with low delayed recall performance (based on a word list) were categorized as aMCI. The diagnosis of AD was made based on the National Institute of Neurological and Communicative Disorders and Stroke-Alzheimer's Disease and Related Disorders Association (NINCDS-ADRAD) criteria. HCs were drawn from volunteers with no history of neurological disease or disorders and no history of brain trauma. This study was approved by the Institutional Review Board at Samsung Medical Center and was performed in accordance with the Declaration of Helsinki. Written informed consent was provided by both participants and/or caregivers.

**Table 1:** **Demographics**.

	**HC**	**aMCI**	**AD**	***p*-value**
**ALZHEIMER'S DISEASE**				
*N*	20	39	24	
Age	71.6 ± 4.9	74.7 ± 5.2	74.9 ± 5.1	0.054
Sex	8M:12F	16M:23F	9M:15F	0.96
CDR		0.5	0.5	–
MMSE		26.2 ± 2.1	21.3 ± 3.8	<0.0001
SVLT_Delayed		1.5 ± 1.5	0.9 ± 1.8	0.875
RCFT_Delayed		5.8 ± 4.1	2.3 ± 2.64	0.0005

Resting state MRI scans were obtained using a 3.0T scanner (Model: Philips Intera Achieva, Phillips Healthcare, Netherlands) for 5 min. Scans involved the acquisition of 35 axial slices using a gradient echo planar imaging pulse sequence as follows: TR = 3,000 ms; TE = 35 ms; FOV = 220 mm; voxel size (RL, AP) = 2.875 × 2.875 mm with a slice thickness of 4 mm. Additionally, T1- weighted anatomical images were obtained for each participant using the following sequence: TR = 1,114 ms; TE = 10 ms; FOV (RL, AP, FH) = 220 × 220 × 132 mm; and REC voxel size = 0.43 × 0.43 × 0.43 mm.

Reproducibility analysis was done on a separate dataset. Resting dataset from 93 healthy controls with no history of neurological diseases was gathered. Four subjects were discarded due to MRI quality. Subjects were interviewed by experienced psychiatrists using The Structured Clinical Interview for the Diagnostic and Statistical Manual of Mental Disorders, Fourth edition, Axis I (SCID-I). This study was approved by the Institutional Review Board at Seoul National University Hospital and was performed in accordance with the Declaration of Helsinki. Written informed consent was provided by participants and/or caregivers/guardians.

Resting-state MRI scans were acquired using a 3.0T scanner (Model: Siemens Trio, Siemens Healthcare, Erlangen, Germany) for 8 min and 45 s. Scans involved the acquisition of 35 axial slices using a gradient echo planar imaging pulse sequence as follows: TR = 3,500 ms; TE = 30 ms; FOV = 240 mm; voxel size (RL, AP) = 1.9 × 1.9 mm; and slice thickness = 3.5 mm. Additionally, T1-weighted anatomical images were obtained for each participant using the following sequence: TR = 1,890 ms; TE = 1.89 ms; FOV = 240 mm; and voxel size (RL, AP, FH) = 1.0 × 1.0 × 1.0 mm.

### MRI data pre-processing

Pre-processing of fMRI and structural MRI data was performed using MRIcron (http://people.cas.sc.edu/rorden/mricron/index.html) and the FMRIB Software Library (FSL, www.fmrib.ox.ac.uk/fsl/). MRIcron converted the raw fMRI images to a compressed FSL format. Image pre-processing consisted of skull stripping using the Brain Extraction Tool (BET), slice timing correction, temporal high-pass filter (Gaussian-weighted least-squares line fitted with sigma = 100.0 s), MCFLIRT motion correction, spatial smoothing (using a Gaussian kernel of FWHM 4 mm) and global signal regression. FLIRT (FMRIB's Linear Image Registration Tool) was used to register and normalize the images to the Montreal Neurological Institute (MNI) template (2-mm resolution). Group measures of head movement were measured using the FSL Motion Outliers with framewise displacement (FD). White matter signals were identified from ICA and CSF signals were identified by averaging time series from the left and right ventricles. Both signals were regressed out using partial correlation during subsequent analyses.

### MRI data processing

Group MELODIC ICA was performed with a 25 component selection. Major networks were identified within subjects using FSL's MELODIC software. Four major networks were identified: the default mode network (DMN), the left and right fronto-parietal networks (FPNL and FPNR), and the salience network (SAL). Each network was then separated into various brain regions and masks were created for each region. Resulting networks from ICA were transformed to MNI space. The regions selected for each network were based on distinct and commonly reported regions in each resting network (Damoiseaux et al., 2006; Watanabe et al., 2013; Betzel et al., 2014; Chan et al., 2014). Major regions in each network were identified in the corresponding ICA network and masks were created over each of these regions. Excess voxels in each region were trimmed by hand. Regional masks created for the DMN include the prefrontal cortex, posterior cingulate cortex (PCC), the left and right parietal lobes (PLL, PLR), and the left and right hippocampus (HCL, HCR). The regions created for the FPNL and FPNR were the left and right frontal lobe, the anterior cingulate cortex (ACC), the PCC, the PLL, PLR, and the corresponding left or right occipital temporal cortex. For the SAL network, regions identified were the ACC, the left and right superior frontal gyrus, and the left and right inferior frontal gyrus. Time-series were extracted for each voxel within each region.

### Node identification

**Figure 1:**
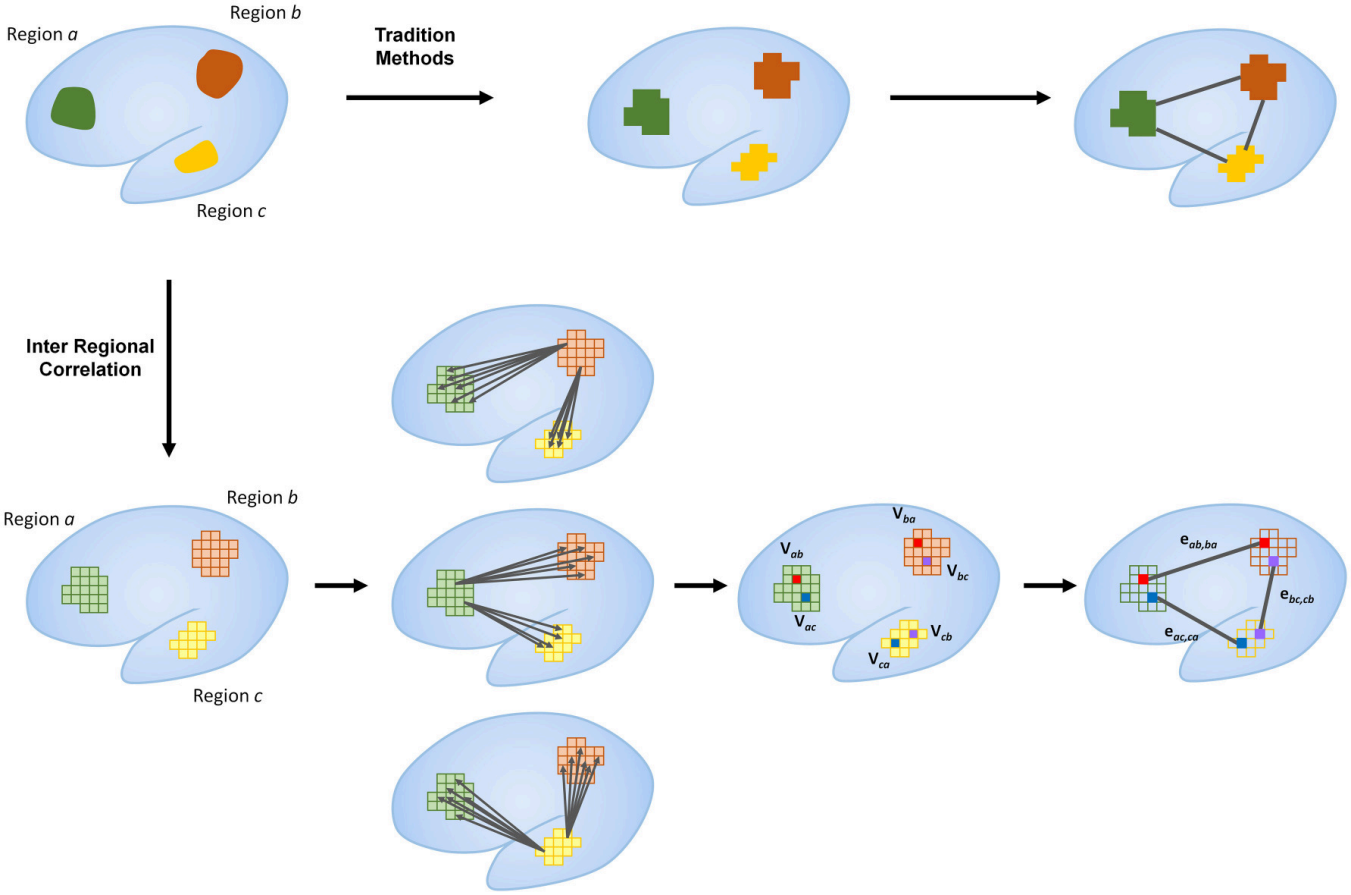
**Outline of proposed method compared to traditional methods**. Traditional methods typically use large regions as nodes for network analysis. Regional correlation identifies specific nodes within each region that represent relative hubs to other regions in the same network. This is calculated by the identification of the voxel in a given region with the highest correlation to another region. Identification of these hub voxels as nodes within each network region allows for more detailed representations of network connectivity. The current figure shows only primary connectivity. Further breakdown of connectivity measures are outlined in Figure 2.

Node identification was defined by the voxel with the highest correlation to another region (Figure 1). Voxel by voxel correlations were calculated with a threshold at *r* > 0.25. This threshold is selected based on parameters set in global brain connectivity (Buckner et al., 2009; Cole et al., 2010). The total correlation of every voxel in a given region was calculated by summing the correlation to all the voxels in another region. The voxel with the highest total correlation was identified and selected as the representative node to the other network region. The time-series for that voxel was extracted for subsequent analyses.

#### Notation

**Figure 2:**
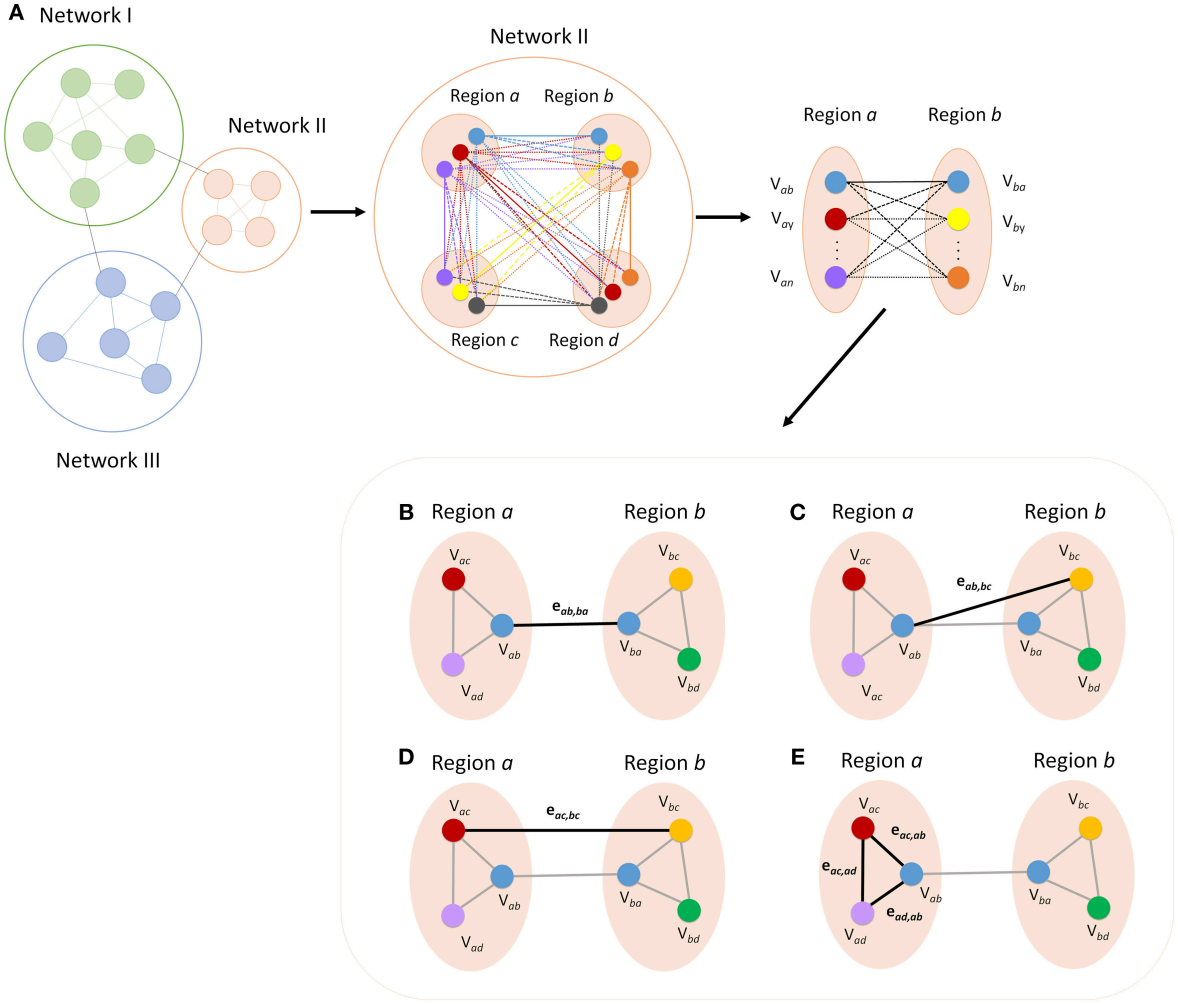
**Breakdown of regional areas from major networks allows for the calculation of connectivity between specific nodes within each network**. The figure shows a representation of how more detailed measures of functional connectivity can be derived **(A)**. All connections within a given network can be broken down and defined according to how each node was defined. Functional definition allows for labeling of each ROI and edge for categorization into specific types of nodes and connections. Primary connectivity (R_1_) is the connection between the two nodes that were defined by maximum connectivity between the opposite region **(B)**. A primary to secondary connection (R_12_) is the connectivity between a primary node and a node that is a primary node to another region **(C)**. Secondary connections (R_2_) are the connections between secondary nodes in the two regions **(D)** and intra-regional connections (R_I_) are the connections between the nodes in a given region **(E)**.

Definitions for all nodes are defined in the context from one region to another. Each node will be notated as V_*xy*_, where *x* is the region where the node is located and *y* is the target region used to identify the node. Connections or edges will be notated as e_*kl, mn*_, which represents the connection between V_*kl*_ and V_*mn*_ (Figure 2). The unique process in which these nodes are identified provides new definitions of specific nodes and edges. For example, the nodes and connections between two regions (regions *a* and *b*) can be characterized based on how they were defined (Figure 2A). These nodes, derived from voxel to voxel correlations to these regions, are defined as primary nodes (V_*ab*_ and V_*ba*_), and the connection between these two is defined as a primary connection (R_1_, Figure 2B). If we continue to examine the connectivity in the context from region *a* to region *b*, then the connections between the nodes derived from connectivity to other regions (regions *c* and *d*) are labeled secondary nodes (V_*ac*_, V_*ad*_, V_*bc*_, and V_*bd*_). The connection between a primary node and secondary node can be called a primary to a secondary connection (R_12_, Figure 2C), and a connection between secondary nodes can be called a secondary connection (R_2_, Figure 2D). Finally, connections between nodes in the same functional region are defined as intra-regional connections (R_I_, Figure 2D). If the context of which brain regions are being investigated is changed, the definition of the nodes will also change. For example, if we instead look at the connections between regions *a* and *c*, the primary nodes become V_*ac*_ and V_*ca*_, where, in the context of looking at connectivity between *a* and *b*, these were secondary nodes. It is important to note that the while the definition of nodes will change with the context, the definitions of the connections or edges will not change as the context that the connection represents is always fixed. These new definitions allow for new measures of connectivity that can be used to measure the strength of network connectivity and to characterize and identify patterns of changes in network connectivity.

### Measures of correlation

**Figure 3:**
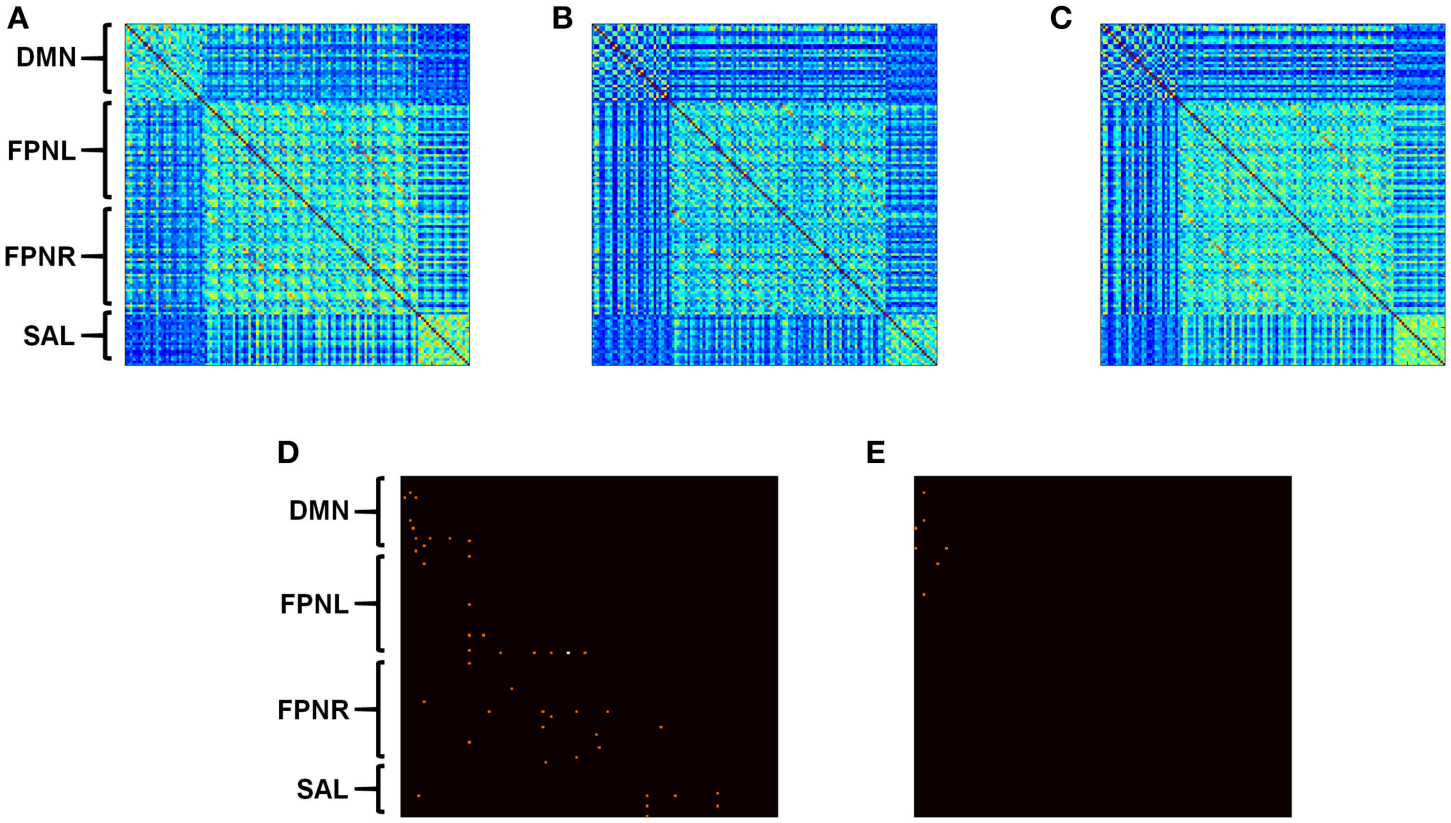
**Calculated correlation with AD progression**. Average functional connectivity was calculated for all nodes in HCs **(A)**, aMCI **(B)**, and AD **(C)**. Connectivity matrices represent pairwise connectivity between all derived nodes. Significant differences in correlation between HCs and disease groups are shown for aMCI **(D)** and AD **(E)**. Subject group sizes included 20 HCs, 39 aMCI subjects and 24 AD patients. Connections that are significant after correction are shown in white (α < 0.05). Uncorrected *p* < 0.001 is shown in orange.

Tradition measures of correlation (ROI to ROI) are calculated and shown using a correlation matrix (Figure 3). Correlations values are obtained for each subject and averaged for each subject group. Comparison of correlation values between AD groups and healthy controls was performed using an unpaired *t*-test. Unequal variances were accounted for using Satterthwaite's approximation. Statistical significance are reported using *p*-values. Because of the number of connections being compared, significance for correlation matrices are reported using both an uncorrected *p*-value of less than 0.001, in addition to FDR corrected connections (α < 0.05).

For each network, the measures of correlation are calculated between regions and then averaged across the whole network. In addition, regions of the DMN were further broken down and analyzed for each measure based on overall connectivity to each region. The equations below represent the calculated connectivity between two specific regions within a given network.

#### Primary connectivity (R_1_)

Connections between nodes derived from the correlation to analogous regions will be defined as primary connections. For example, if a node in region *a* was determined by connectivity to region *b* (V_*ab*_) and another node in region *b* was determined by connectivity to node *a* (V_*ba*_), then the connectivity between the two nodes is a primary connection. The equation for the primary connection can be determined by:

(1) $${\text{R}}_{1\text{ab}}={\text{e}}_{ab,ba}$$

#### Primary to secondary connectivity (R_12_)

However, more than one correlation is calculated between regions *a* and *b*. Correlations calculated between the primary node V_*ab*_ and the remaining nodes in region *b* are defined as primary-secondary connections. Primary to secondary connectivity between two specific nodes is defined as the average of all such connections. The equation can be expressed as:

(2) $${\text{R}}_{12\text{ab}}=\frac{\left(\sum_{i\;=\;1}^{N-1}{\text{e}}_{ab,bi}\;+\;\sum_{i\;=\;1}^{N-1}{\text{e}}_{aj,ba}\right)}{2(\text{N}-2)}$$

where *N* is defined as the total number of regions in the network. Since no node is determined from the connectivity to the region in which it resides (i.e., V_*aa*_), the summation must follow that when *i* ≥ *a*, then *i* = *i* + 1, and when *j* > = b, then *j* = *j* + 1.

#### Secondary connectivity (R_2_)

Secondary connections are the remaining connections calculated between nodes derived independently from the connectivity between the two regions. To put it simply, secondary connections can be calculated by the total correlation between the two regions minus the R_1_ and R_12_ connections. The equation can be expressed as:

(3) $${\text{R}}_{2\text{ab}}=\frac{\left(\sum_{i,\;j\;=\;1}^{N-1}{\text{e}}_{ai,bj}-\left(\sum_{i\;=\;1}^{N-1}{\text{e}}_{ab,bi}+\sum_{j\;=\;1}^{N-1}{\text{e}}_{aj,ba}\right)-e_{ab,ba}\right)}{{\left(\text{N}-2\right)}^{2}}$$

where N is defined as the total number of nodes in the network. Again, since no node is determined from the connectivity to the region in which it resides, the summation must follow that when *i* ≥ *a*, then *i* = *i* + 1, and when *j* > = *b*, then *j* = *j* + 1.

#### Intra-regional connectivity (R_I_)

Intra-regional connectivity is defined as the correlations between ROIs within the same region of a given network. The value is calculated as:

(4) $${\text{R}}_{\text{Ia}}=\sum_{i\;=\;1,\;j=i\;+\;1}^{N-1}{\text{e}}_{i,j}$$

where *N* is defined as the total number of nodes in the network. Since no node is determined from the connectivity to the region in which it resides, the summation must follow that when *i* ≥ *a*, then *i* = *i* + 1, and when *a* ≥ *b*, then *j* = *j* + 1.

Comparison of newly derived measures of connectivity (R_*I*_,R_1_, R_12_, and R_2_) was calculated between groups using ANOVA with *post-hoc* analysis. For all analysis involving new measures. Significance is reported with a corrected value of α < 0.05 using Benjamini-Hochberg correction. Corrections are done within similar measures of connectivity.

Following the extraction of time-series from brain regions using FSL, all calculations and subsequent analyses were performed using in house coding in MATLAB. Codes and masks used in this study are available upon request.

### Reproducibility of ROIs

The reproducibility of ROIs was evaluated based on various masks from the DMN. For this analysis, the regions that were targeted for analysis was the PCC, PFC, PLL, and PLR regions of the DMN. The original masks used in the AD analysis were either eroded, adjusted or inflated to varying degrees (Table 2) to simulate different ICA results. Eroded masks represent possible ICA results that can be significantly smaller than the regions defined in our study, and inflated masks represent possible ICA results that can be significantly larger. In addition, ICA was performed on the reproducibility dataset using a 25 component decomposition to create a new set of masks. The DMN was identified and a new set of masks were created for the regions mentioned above (Supplementary Figure 1). Inter-regional correlation analysis was performed for each set of masks, and the extracted time-series were then compared with the time-series obtained from using the original masks.

**Table 2:** **Size of masks (# of voxels) used to test reproducibility of node identification**.

	**Original**	**Eroded**	**Adjusted**	**Inflated**	**New**
**DMN ROIs**					
PCC	6490	M1: 4080 M2: 2354	M1: 6349 M2: 6254	M1: 9523 M2: 13096	M1: 5687
PFC	6944	M1: 4538 M2: 2692	M1: 6814 M2: 6728	M1: 9909 M2: 13443	M1: 5823
PLL	4957	M1: 2830 M2: 1309	M1: 4834 M2: 4745	M1: 7591 M2: 10723	M1: 3801
PLR	5101	M1: 2947 M2: 1371	M1: 4981 M2: 4888	M1: 7780 M2: 10999	M1: 3265

## Results

### Connectivity matrix

Standard correlation analysis revealed decreases in connectivity between the edges in all networks when comparing subjects with aMCI to HCs (Figure 3D, *p* < 0.001). When comparing connectivity between AD patients and HCs, only connectivity in the DMN and the connections between the DMN and FPNL network showed significant decreases (Figure 3E, *p* < 0.001).

### Overall network connectivity

**Figure 4:**
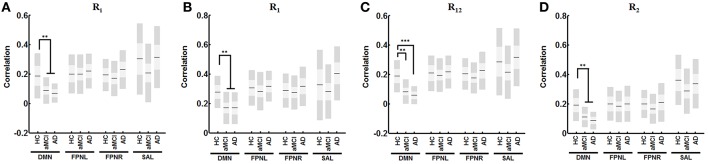
**Change in derived functional measures for each network with AD progression**. Analysis reveals significant decreased correlation with for R_I_ **(A)** R_1_ **(B)**, R_12_ **(C)**, and R_2_ **(D)** in the DMN with no changes in FPNL, FPNR, and SAL connectivity. Graph shows mean, SD, and 95% confidence intervals. Lines with asterisks show which groups demonstrated statistical differences: ^**^α < 0.01, ^***^α < 0.001. Only connections that are significant after FDR correction for multiple comparison are shown (α < 0.05).

Newly defined measures showed distinct changes in network connectivity. Analysis showed progressively decreased connectivity in the DMN with all measures of connectivity in aMCI. Analysis showed significantly decreased R_1_ connectivity (Figure 4B, α = 0.007), R_12_ connectivity (Figure 4C, α < 0.001), R_2_ connectivity (Figure 4D, α = 0.005) and R_I_ connectivity (Figure 4A, α = 0.010 in aMCI compared to HCs. When comparing AD patients to HCs, the analysis showed a significant decrease in R_1_ connectivity (Figure 4B, α = 0.020), R_12_ connectivity (Figure 4C, α < 0.001), R_2_ connectivity (Figure 4D, α < 0.001), and R_I_ connectivity (Figure 4A, α = 0.004). The FPNL and FPNR networks showed no significant changes in aMCI or AD.

### Connectivity of each region in the DMN

**Figure 5:**
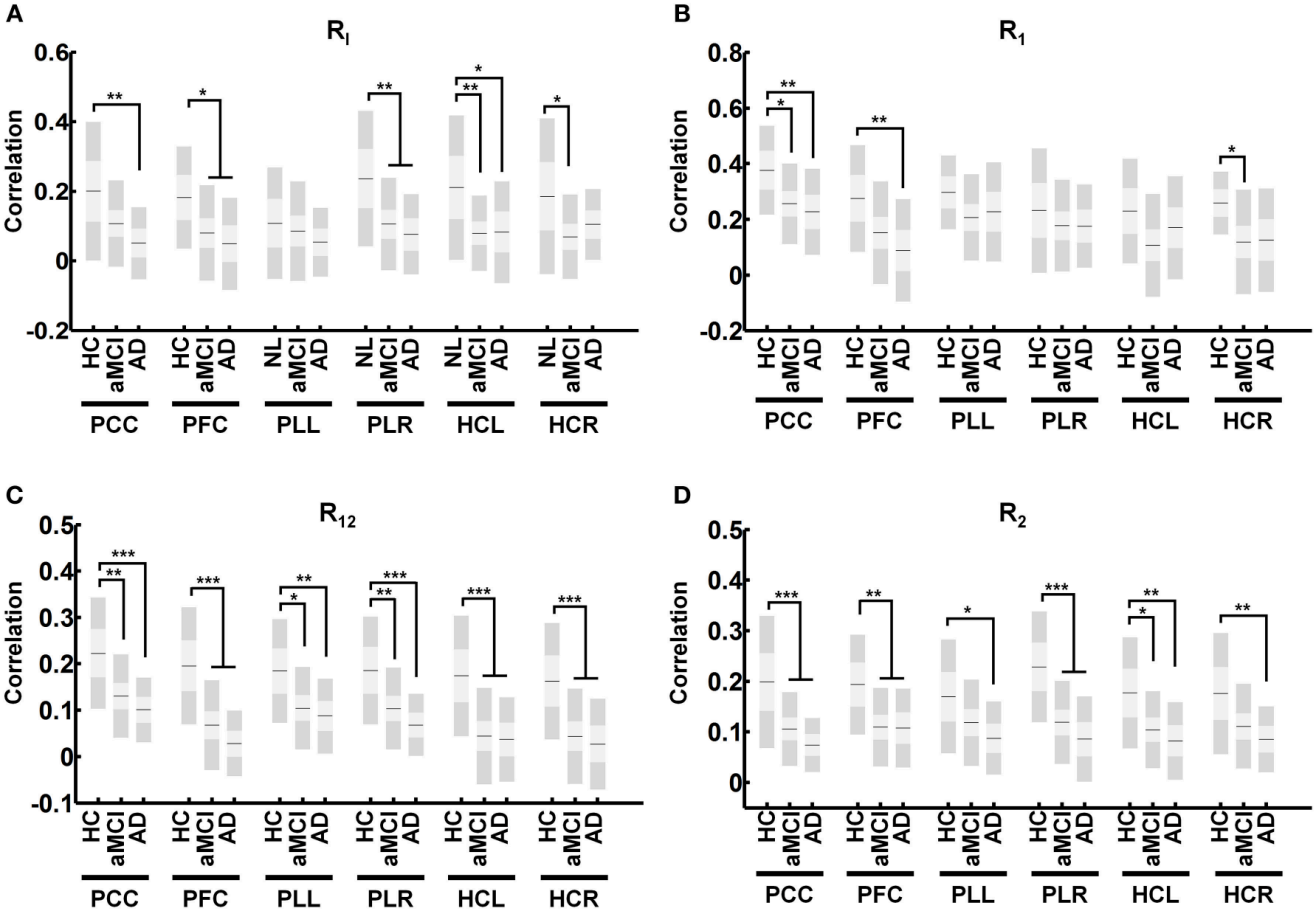
**Average connectivity for each region of the DMN for derived measures**. Average calculated connectivity for R_I_ **(A)** R_1_ **(B)**, R_12_ **(C)**, and R_2_ **(D)** in each region shows R_12_ and R_2_ connectivity to be universally affected in regions of the DMN while R_1_ connectivity remains relatively intact. Graph shows mean, SD, and 95% confidence intervals. Lines with asterisks show which groups demonstrated statistical differences: ^*^α < 0.05, ^**^α < 0.01, ^***^α < 0.001. Only connections that are significant after FDR correction for multiple comparison are shown (α < 0.05).

In the DMN, R_I_ connectivity showed significant decreases in aMCI subjects in the PFC (Figure 5A, α = 0.046), the PLR (Figure 5A, α = 0.009), the HCL (Figure 5A, α = 0.009) and HCR (Figure 5A, α = 0.029). AD patients showed decreases in significant R_I_ connectivity in the PCC (Figure 5A, α = 0.004), PFC (Figure 5A, α = 0.012), PLR (Figure 5A, α = 0.003), and HCL (Figure 5A, α = 0.031). R_1_ connectivity shows significant decreases in aMCI subjects in the PCC (Figure 5B, α = 0.026), and HCR (Figure 5B, α = 0.022). AD patients showed significant decreases in R_1_ connectivity in the PCC (Figure 5B, α = 0.009), and PFC (Figure 5B, α = 0.008). R_12_ shows significant decreases in aMCI subjects in the PCC (Figure 5C, α = 0.003), PFC (Figure 5C, α < 0.001), PLL (Figure 5C, α = 0.013), PLR (Figure 5C, α = 0.007), HCL (Figure 5C, α < 0.001) and HCR (Figure 5B, α < 0.001). AD patients showed decreases in significant R_12_ in the PCC (Figure 5C, α < 0.001), PFC (Figure 5C, α < 0.001), PLL (Figure 5C, α = 0.005), PLR (Figure 5C, α < 0.001), HCL (Figure 5C, α < 0.001), and HCR (Figure 5C, α < 0.001). Finally, R_2_ connectivity shows significant decreases in aMCI subjects in the PCC (Figure 5C, α < 0.001), PFC (Figure 5C, α = 0.002), PLR (Figure 5C, α < 0.001), and HCL (Figure 5C, α = 0.014). AD patients showed decreases in significant R_2_ in the PCC (Figure 5C, α < 0.001), PFC (Figure 5C, α = 0.006), PLL (Figure 5C, α = 0.018), PLR (Figure 5C, α < 0.001), HCL (Figure 5C, α = 0.003), and HCR (Figure 5C, α = 0.007).

### Reproducibility analysis

Comparison of extracted time-series using various masks for analysis shows small differences based on how masks are defined. Analysis shows close to no change with masks that are varying in ~100–200 voxels (Table 3). Differences in extracted time series become more evident with larger differences in masks sizes however correlation still remains relative high for all different masks. Time-series which are extracted from masks that are derived entirely from separate datasets still show high correlation among one another (Table 3). These results are consistent across all individual regions which are tested in our analysis (Table 4).

**Table 3:** **Average correlation of time-series identified from different masks with the original time-series**.

**Eroded**	**Adjusted**	**Inflated**	**New**
**AVERAGE CORRELATION**			
M1: 0.87 ± 0.16 M2: 0.78 ± 0.18	M1: 0.98 ± 0.07 M2: 0.98 ± 0.08	M1: 0.89 ± 0.17 M2: 0.82 ± 0.20	M1: 0.81 ± 0.19

**Table 4:** **Average correlation of time-series extracted from each region of the DMN using different masks with the original time-series**.

**DMN ROIs**	**Eroded**	**New**	**Inflated**	**New**
PCC	M1: 0.90 ± 0.13 M2: 0.83 ± 0.16	M1: 0.99 ± 0.05 M2: 0.99 ± 0.06	M1: 0.91 ± 0.15 M2: 0.84 ± 0.18	M1: 0.81 ± 0.17
PFC	M1: 0.87 ± 0.17 M2: 0.79 ± 0.21	M1: 0.98 ± 0.08 M2: 0.98 ± 0.09	M1: 0.87 ± 0.19 M2: 0.81 ± 0.21	M1: 0.78 ± 0.21
PLL	M1: 0.86 ± 0.17 M2: 0.76 ± 0.20	M1: 0.98 ± 0.08 M2: 0.98 ± 0.09	M1: 0.89 ± 0.16 M2: 0.82 ± 0.21	M1: 0.82 ± 0.18
PLR	M1: 0.86 ± 0.17 M2: 0.75 ± 0.16	M1: 0.98 ± 0.07 M2: 0.98 ± 0.07	M1: 0.88 ± 0.18 M2: 0.81 ± 0.21	M1: 0.82 ± 0.19

## Discussion

### Identification of functional nodes using regional correlation

The new measures derived in this method provide an intuitive breakdown of the connections that exist in a network. Currently, node definitions only allow for comparisons of specific connectivity (Figure 3). These offer limited information other than the presence of a disconnection between two nodes. However, if we are able to define and group certain connections, it allows for a deeper understanding of network characteristics in the brain. Therefore, the exact significance behind these definitions should be discussed. R_1_ connections can represent the strength of direct connections between two regions of a network. R_12_ and R_2_ connections represent connections between relative hub regions that may not be directly connected. Finally, R_I_ connectivity represents the local connectivity within each region or intra-regional connectivity. Further studies are needed to confirm the biological basis behind these measures.

In our analysis, R_1_ and R_I_ connections showed higher calculated correlations than R_12_, and R_2_ connections (Figures 4, 5). This was expected based on the significance behind each type of connection. R_1_ connections represented connectivity between ROIs selected due to their high correlation to the analogous region. In contrast, R_12_ connections represented connectivity between ROIs where one was not a representative node to the other region and as such had lower calculated correlations. Finally, because R_2_ is calculated between two secondary ROIs, they showed the lowest correlation.

This method is not the first to explore methods utilizing the idea of identifying voxels with high connectivity for subsequent analysis. Several studies advocate using various voxel-wise approaches and highlight the advantages of identifying key hub regions over large regional parcellations (van den Heuvel et al., 2008; Cole et al., 2010; Liang et al., 2015; Rajtmajer et al., 2015; Lee K. et al., 2016). Previous methods such as global brain connectivity (GBC) analyze the correlations of every voxel with every other voxel in the brain to determine whole brain connectivity (Cole et al., 2010). This method is useful for identifying globally connected regions, but it cannot distinguish the local hubs that play a significant role in specific networks. Another method called inter-voxel cross-correlation (Golestani and Goodyear, 2011) identifies highly correlated voxels between two regions but uses the ROIs for whole brain analysis. Our method is distinguished from these methods in that we are able to define and characterize specific types of connections which exist in a connectome.

### Functional changes in Alzheimer's disease

AD is a progressive neurodegenerative disorder characterized by the formation and accumulation of amyloid plaques and tau protein tangles in the brain (Querfurth and LaFerla, 2010) Previous studies with resting state fMRI have revealed that the DMN is particularly vulnerable in AD (Buckner et al., 2005; Lee E. S. et al., 2016). In this study, analysis comparing calculated connectivity with correlation matrices alone did not reveal any discernable pattern in connectivity changes (Figure 3). Overall we observe general decreases in network connectivity in aMCI (Figure 3D) however showed only decreased connectivity in the DMN and connectivity to the DMN and FPNL for AD (Figure 3E). Only one connection was significant after multiple comparison correction. The difficultly of interpreting these results highlights the need for new measures which can, to a degree, organize and consolidate correlation measures for better interpretability. Analysis using the new measures defined in this study showed significant decreases in connectivity with R_1_, R_12_, R_2_, and R_I_ in the DMN. We showed a progressive decrease in connectivity in the DMN, no changes in the FPNL and FPNR networks and fluctuating changes in the SAL network (Figure 4). These results were consistent with previous studies that showed that changes in functional connectivity were shown most distinctly in the DMN (Buckner et al., 2005; Seeley et al., 2009; Brier et al., 2014; Crossley et al., 2014; Sohn et al., 2014; Toussaint et al., 2014). In our study, we observed decreased connectivity in the DMN network beginning in aMCI.

From a network perspective, AD pathology is thought to exemplify the “disconnection” hypothesis (Brier et al., 2014). The formation of amyloid deposition and tau proteins in key regions of the brain result in loss of synapses in the brain, which leads to the loss of functional connectivity (Small, 2008; Lim et al., 2014; Myers et al., 2014; Chung et al., 2016). Studies have revealed that structures or regions that compose the DMN are highly susceptible to AD pathology (Buckner et al., 2005; Querfurth and LaFerla, 2010). As a result, the deterioration in the functional connectivity of the DMN has been highly reflective of AD pathology. Our study showed a decrease in all measures of connectivity in only the DMN. We propose that the results obtained in our study accurately reflect the pathological effects of AD on functional connectivity in the brain. The significant decreases in R_1_, R_12_, R_2_, and R_I_ connectivity functional changes occur in AD at all levels. Therefore, we propose that the network deterioration caused by the amyloid deposition and tau proteins in AD is a network-wide phenomenon that targets all types on connections within the DMN.

Additional analysis of the regions within in the DMN showed a detailed picture of the vulnerability of each region in AD. Numerous models have been proposed in network degeneration (Zhou et al., 2012; Brier et al., 2014; Fornito et al., 2015), however many of these models do not fully account for the observed changes in functional connectivity. Our results supports a model which proposes that while specific brain regions are affected by AD pathology, they continue to communicate in a disrupted manner (Brier et al., 2014). Our results show that not all regions are affected equally. Significant decreases in R_1_ connectivity only occurred in the PCC, PFC, and HCR regions (Figure 5B). However, with the exception of the PLL in R_I_ connectivity, all other measures showed significant decreases in all regions of the DMN (Figure 5). These results show that R_1_ connectivity is relatively unaffected compared to other measures, suggesting that a major contributor to deficits in intrinsic connectivity of the DMN can be attributed to decreased connectivity to secondary hubs (R_12_, R_2_ connectivity). The detailed information we obtain from breaking down and defining specific measures of connectivity illustrates the strengths our method has in uncovering functional changes which occur in neurological diseases.

### Limitations and future directions

**Figure 6:**
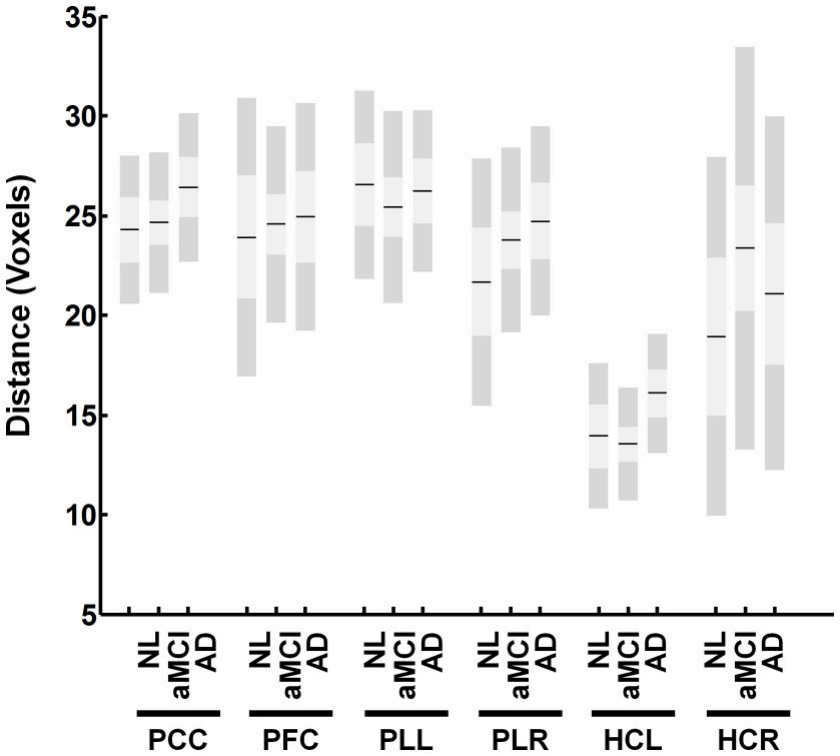
**Distance between identified nodes for the DMN**. The figure shows the average distance in voxels between identified nodes within each region. The figure shows no significant differences between groups.

One important issue is the possibility that the same voxel can be selected as a primary node to multiple regions because the resulting calculated correlation between the two voxels will be one. Additionally, nodes that are spatially too close to one another demonstrate high connectivity. This phenomenon could be the cause of higher intra-regional correlation obtained in our study for HCs compared to AD. We showed that the actual spatial differences between intra-regional ROIs are similar between different AD and HCs (Figure 6). Additionally, no specific measures for between network (extrinsic) connectivity, were defined in this paper. However, it would not be difficult to develop such measures for further analysis.

Finally, this method is dependent on networks derived from ICA decomposition. Since this method begins by first identifying the main regions of networks, only major networks that have been shown to be consistently reproduced were used (Beckmann et al., 2005; Abou Elseoud et al., 2011; Chou et al., 2012). Even so, ICA iterations can produce slightly different results from the same dataset (McKeown et al., 2003). We show that small variations in region size have very little effect on extracted time series (Tables 3, 4). Even large variations in region size reveal a relatively small effect on the extracted time-series, showing that this method will be robust for reasonable differences in ICA results. Variations in ICA results can be eliminated by using standardized functional templates. It is possible to select these regions from anatomical atlases; however, the functional relevance of deriving such ROIs should be considered. The same is true with different high-resolution functional parcellation techniques (Sohn et al., 2012; Igelström et al., 2015; Wang et al., 2015; Moher Alsady et al., 2016). While these techniques can provide more regions for analysis, the significance behind new network measures calculated using these techniques might not be as evident. New emerging techniques may provide a means for organizing these parcellations (Power et al., 2011; Li and Wang, 2015; Yoo et al., 2017).

## Conclusion

In this study, we used a regional correlation based method for node identification to discern detailed changes in functional connectivity in AD. Specifically, we found that while all connections within the DMN were affected in AD, specific regions were more affected in certain measures. The mapping of functional connectomes provides a powerful tool for the mapping, tracking and prediction of patterns of changes in connectivity with the progression of brain disease. The unique way in which nodes and ROIs are defined in this study allows for the use of new descriptive features to analyze changes in functional connectivity. We show that by using new measures, we can contribute new information regarding changes in connectivity that may reflect the underlying pathology and mechanisms behind network deterioration in neurological diseases.

## Author contributions

WS: Designed and planned study, coded program, analyzed data, wrote manuscript. TL: Contributed to methods and discussion. KY: Contributed to methods, analysis, and discussion. MK: Contributed to methods and discussion. JH: Contributed to methods and discussion. JY: Contributed to methods and discussion. YY: Data organization. SS: Collected data, contributed to discussion. DN: Collected data, contributed to discussion. YJ: Co-corresponding author. Designed and planned study. JK designed and planned study.

### Conflict of interest statement

The authors declare that the research was conducted in the absence of any commercial or financial relationships that could be construed as a potential conflict of interest.

## Supplementary material

Supplementary Figure 1 **Different masks used for reproducibility analysis**. Figure shows two sets of mask that were used to test reproducibility for inter-regional correlational analysis. The red mask is the original mask used in analysis of AD subjects and the green masks is a new set of masks which are derived from ICA analysis on a separate dataset.
