# Supplementary figures and images for: Node Identification Using Inter-Regional Correlation Analysis for Mapping Detailed Connections in Resting State Networks
Source: Front Neurosci. 2017 May 1;11:238. doi: 10.3389/fnins.2017.00238 (PMC5410606; doi:10.3389/fnins.2017.00238)

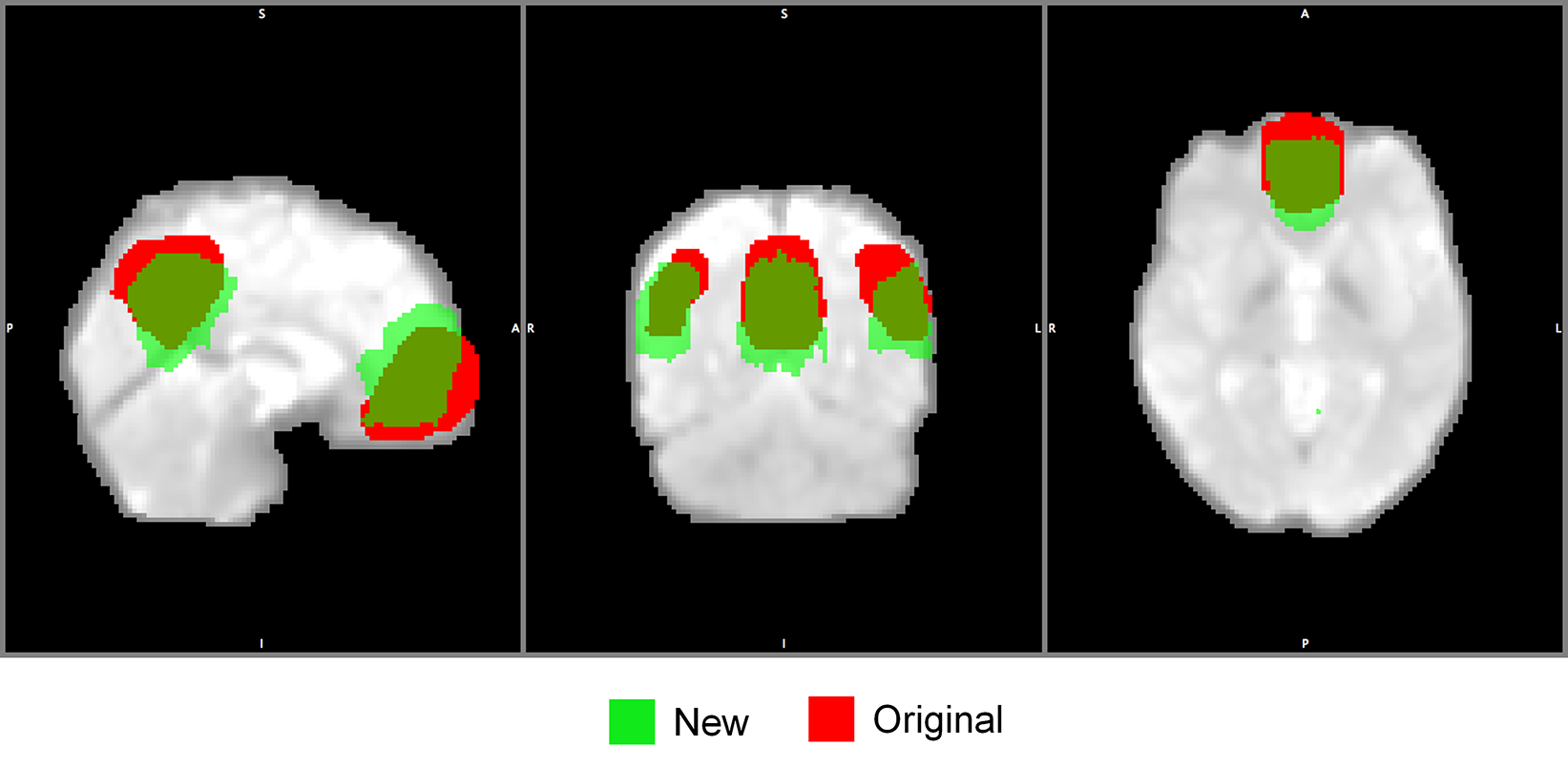

Supplement: Supplementary Figure 1 — Different masks used for reproducibility analysis. Figure shows two sets of mask that were used to test reproducibility for inter-regional correlational analysis. The red mask is the original mask used in analysis of AD subjects and the green masks is a new set of masks which are derived from ICA analysis on a separate dataset. [file Image1.TIF]
